# The increased inter‐brain neural synchronization in prefrontal cortex between simulated patient and acupuncturist during acupuncture stimulation: Evidence from functional near‐infrared spectroscopy hyperscanning

**DOI:** 10.1002/hbm.26120

**Published:** 2022-10-18

**Authors:** Li Chen, Yuzhu Qu, Jingya Cao, Tianyu Liu, Yulai Gong, Zilei Tian, Jing Xiong, Zhenfang Lin, Xin Yang, Tao Yin, Fang Zeng

**Affiliations:** ^1^ Acupuncture and Tuina School Chengdu University of Traditional Chinese Medicine Chengdu Sichuan China; ^2^ Acupuncture and Brain Science Research Center Chengdu University of Traditional Chinese Medicine Chengdu Sichuan China; ^3^ Sport and Healthy School Chengdu University of Traditional Chinese Medicine Chengdu Sichuan China; ^4^ Department of Neurology Sichuan Provincial Rehabilitation Hospital Chengdu Sichuan China; ^5^ Rehabilitation Medicine Center and Institute of Rehabilitation Medicine, West China Hospital Sichuan University Chengdu Sichuan China; ^6^ Health and Rehabilitation School Chengdu University of Traditional Chinese Medicine Chengdu Sichuan China

**Keywords:** acupuncture, fNIRS, hyperscanning, inter‐brain neural synchronization

## Abstract

The patient–acupuncturist interaction was a critical influencing factor for acupuncture effects but its mechanism remains unclear. This study aimed to examine the inter‐brain mechanism of patient–acupuncturist dyad during acupuncture stimulation in a naturalistic clinical setting. Seventy healthy subjects (simulated “patients”) were randomly assigned to two groups and received verum acupuncture group or sham acupuncture by one acupuncturist. Functional near‐infrared spectroscopy hyperscanning was used to simultaneously record the neural responses of “patient”–acupuncturist dyad during acupuncture stimulation in each group. The results showed that inter‐brain neural synchronization (INS) in the prefrontal cortex (PFC) of “patient”–acupuncturist dyad was significantly increased during verum but not sham acupuncture stimuli, and positively correlated with the needling sensations of “patients.” Granger causality analysis demonstrated that there were no significant differences in INS direction between the “patient” and the acupuncturist. This study identified the increase of INS between “patient” and acupuncturist, and suggested that PFC was important to the interaction of “patient”–acupuncturist dyad.

## Graphical abstract

This study firstly applied fNIRS hyperscanning to investigate the neurobiological mechanism of “patient”–acupuncturist interaction during acupuncture stimuli in a naturalistic clinical setting. The results identified the increase of INS between “patient” and acupuncturist, and found that PFC was important to the interaction of “patient”–acupuncturist. This study deepen our understanding on the patient–acupuncturist interaction, and provided a novel experimental approach for future studies on acupuncture mechanism.

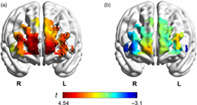

## 1 INTRODUCTION

Acupuncture is one of the most commonly used traditional and complementary therapies worldwide for its efficacy in treating many diseases (Bishop et al., 2021; Zhang et al., 2022). In order to get desired therapeutic effects, the process of traditional Chinese acupuncture therapy requires a high degree of cooperation and a joint experience of needling sensation between the patient and acupuncturist. Both ancient literature and modern research confirmed that patient–acupuncturist interaction was a critical influencing factor for acupuncture effects (Anzolin et al., 2020; Ellingsen et al., 2020; Ellingsen et al., 2022; Fei et al., 2022), and the enhanced interaction between acupuncturist and patient was associated with better therapeutic effect (Suarez‐Almazor et al., 2010). The needling sensation is the key to obtaining acupuncture effect and an essential manifestation of patient–acupuncturist interaction. For patients, the needling sensation refers to the local soreness, numbness, distention, heaviness, etc. For the acupuncturist, the needling sensation manifests as a feeling of sinking and tightness in the fingers holding the needle, which allows the acupuncturist to adjust the manipulation of acupuncture.

During acupuncture manipulation, the sensory information induced by acupuncture stimulation including the local sensation of the patient and the sensation of the acupuncturist's fingers, will be processed and integrated in the central nervous system. In the last two decades, neuroimaging techniques had been widely used to investigate the cerebral responses elicited by acupuncture stimulation. The majority of these studies had focused on the processing of acupuncture signals in the brain of patients, while a few studies concerned on the cerebral feedback of the acupuncturist. Furthermore, almost all the studies investigated the single brain of the patient or the single brain of acupuncturist, and only one study examined the bilateral brain interaction between patient and acupuncturist (Ellingsen et al., 2020). By functional magnetic resonance imaging (fMRI) hyperscanning, Ellingsen et al. (2020) found that the inter‐brain neural synchronization (INS) of patient–acupuncturist dyad was increased after the clinical interaction, and the increased INS was correlated with better therapeutic effectiveness. This study is far from the acupuncture practice in a real clinical environment because the acupuncturist and the patient could only lie on the MRI scanning bed separately, while it provides a good example to study the patient–acupuncturist interaction using hyperscanning technique.

Although nearly all neuroimaging techniques can be used for hyperscanning, functional near‐infrared spectroscopy (fNIRS) has been the most widely used hyperscanning modality to monitor the cortical hemodynamics of interpersonal interactions recently (Hamilton, 2021), and seems to be more suitable for exploring the patient–acupuncturist interaction. Compared to electroencephalogram (EEG), fNIRS has a much higher spatial resolution (Wang et al., 2020). Compared to fMRI, fNIRS has a higher temporal resolution which can more quickly capture the momentary changes of acupuncture signal in the brain, and it is more tolerant to movement artifacts, which can better reflect the acupuncturist‐patient interaction in the real‐world environment. Recently, fNIRS has become increasingly popular in acupuncture research (Fernandez Rojas et al., 2019; Ghafoor et al., 2019; Si et al., 2021; Wong et al., 2021), which demonstrated the feasibility and value of using this neuroimaging technique to investigate the mechanism of acupuncture. Using fNIRS hyperscanning, the current study aimed to investigate (1) INS in patient–acupuncturist dyad during acupuncture stimulation and a correlation between INS and needling sensation; (2) the directionality of INS; (3) the differences of INS in patient–acupuncturist dyad between the verum acupuncture stimulation and sham acupuncture stimulation. We hypothesized that INS would be enhanced for verum acupuncture stimulation, in which the patient and acupuncturist had a joint experience of needling sensation, relative to the sham acupuncture stimulation control, in which no such needling sensation had been experienced by patient and acupuncturist. These results will enhance our understanding on the patient–acupuncturist interaction, and provide a new approach to exploring the central mechanism of acupuncture effect.

## 2 MATERIALS AND METHODS

### 2.1 Participants

The study included 70 healthy male subjects (simulated patients) which were recruited at the campus of Chengdu University of Traditional Chinese Medicine (CDUTCM) from July 2021 to June 2022 The participants should meet all the following criteria: (1) were right‐handed and aged from 18 to 40 years, (2) had no history of neurological or psychiatric disorders and were evaluated with comprehensive history taking, physical examinations, and laboratory tests to exclude the potential disease, (3) did not participate in any other clinical trial. These “patients” were randomly assigned to either verum acupuncture group (VAG) or sham acupuncture group (SAG) in a 1:1 ratio.

One age‐matched licensed female acupuncturist with more than 5 years of clinical experience was recruited from the Affiliated Hospital of CDUTCM. This acupuncturist performed acupuncture manipulations on all the 70 subjects, forming 70 “patient”–acupuncturist dyads in total.

All participants signed the informed consent form before the experiment. Ethical approval (No. 2021QKL‐001) was granted by the Institutional Review Board of Affiliated Hospital of CDUTCM. This study was registered at Chinese Clinical Trial Registry (identifier: ChiCTR2100051893).

### 2.2 Experimental procedures and acupuncture manipulation

**FIGURE 1:**
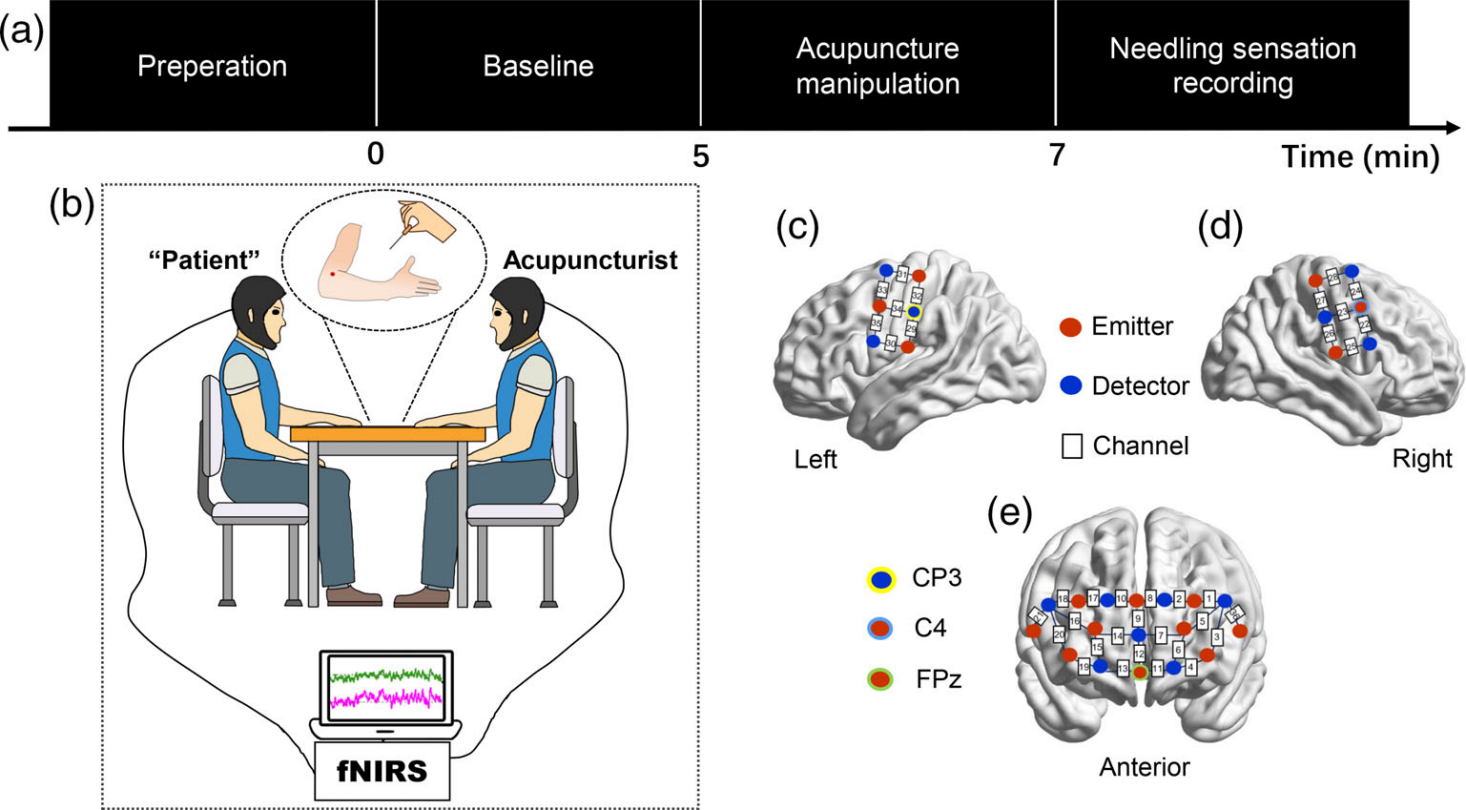
Experimental design and task procedures. (a) Acupuncture experiment paradigm. There are two conditions in the experiment: baseline (5 min) and acupuncture manipulation (2 min). At the end of the experiment, subjects were asked to evaluate the needling sensations. (b) Experimental setup. (c–e) Probe configuration. The integers on the cerebral cortex indicate the recording channels

For VAG, in order to evoke needling sensations and avoid noxious stimulation, the subject's sensitivity and tolerance to needle manipulation were tested before performing acupuncture with fNIRS scanning. The results helped in choosing the depth of needle insertion and the force of needle manipulation to be used for data collection. Throughout the experiment, all dyads sat face‐to‐face during the entire process of the acupuncture manipulation in a quiet room. The whole experimental procedure included the following two sessions (Figure 1a,b). (1) The baseline: all participants were required to keep still and remain as motionless as possible for 5 min, and (2) the verum or sham acupuncture task: verum acupuncture manipulations: the acupuncturist inserted a disposable sterile steel needle (0.20 mm in diameter, 40 mm in length, Hwatuo) into the subject's left *Quchi* (LI‐11, located on the lateral end of the transverse cubital crease, is a commonly used acupoint in clinical practice) for 15–20 mm perpendicularly and performed the even reinforcing‐reducing method with lifting‐thrusting manipulation on the acupoint for 120 s with the depth of 0.3–0.5 cm and frequency of 1 Hz. Sham acupuncture manipulations: the acupuncturist gently tapped the skin over the *Quchi* with a size 5.88 von Frey monofilament at the same frequency (i.e., 1 Hz) as the verum acupuncture stimuli for 120 s. Since verbal communication was not allowed during this session, the subject could show his strong discomfort by pressing the button with his right hand. At the end of the experiment, needling sensation questionnaires were given to the “patients” to evaluate their needling sensations during acupuncture task session.

### 2.3 Needling sensations evaluations

The Chinese version Massachusetts General Hospital Acupuncture Sensation Scale (C‐MASS) (Yu et al., 2012) was used to record the patients' needling sensations. The 10‐point (“0” means none and “10” means unbearable) Visual Analogue Scale was applied to assess the intensity of “patients” needling sensations including soreness, numbness, distention, heaviness, aching, warmth, cold and other sensations.

### 2.4 Functional near‐infrared spectroscopy data acquisition

A multichannel fNIRS system (NIRScout, NIRx Medical Technologies LLC) was employed to measure the oxyhemoglobin (HbO) and deoxyhemoglobin (HbR) concentrations of the dyads simultaneously. The absorption of near‐infrared light was measured at the wavelengths of 785 and 830 nm and the sampling frequency was 3.91 Hz. Considering the key role of sensorimotor cortex (SMC) and prefrontal cortex (PFC) in the central regulation of acupuncture stimuli (Fernandez Rojas et al., 2019; Mawla et al., 2021; Si et al., 2021; Takamoto et al., 2010), we selected bilateral SMC and PFC as regions of interest (ROI). For each participant, two 2 × 3 optode probe sets (3 emitters and 3 detectors probes, 30 mm optode separation) consisting of 7 measurement CHs were placed over the bilateral SMC for each participant (Figure 1c,d). One 3 × 5 optode probe set (8 emitters and 7 detectors probes, 30 mm optode separation) consisting of 22 measurement channels (CHs) was placed over bilateral PFC (Figure 1e). According to the International 10–20 system of electrode placement, the lowest probes were positioned along the Fp1‐Fp2 line, with the middle optode placed on the frontal pole middle point (Fpz). In addition, the middle probe of the probe set was aligned precisely along the sagittal reference curve. The corresponding anatomical positions of fNIRS CHs were measured by an electromagnetic 3D digitizer system (PATRIOT; Polhemus) and registered on the Montreal Neurological Institute (MNI) brain space using a virtual registration method (Table S1).

### 2.5 Functional near‐infrared spectroscopy data analysis

#### 2.5.1 Pre‐processing

This study focused on the changes of the HbO concentration, which was demonstrated to be the most sensitive indicator of changes in the regional cerebral blood flow in fNIRS measurements (Hoshi, 2007, 2016). fNIRS data in the initial 30 s of baseline period were removed and then were pre‐processed by Homer2 Matlab package (homer2_src_v2_8_11022018) as follows. First, the raw optical intensity data were converted to optical density (OD) data. Next, channels with bad signals and long periods of motion artifacts would be removed or recalibrated. Then, a 0.01–0.2 Hz bandpass filter was used to exclude physiological artifacts according to previous fNIRS studies of acupuncture (Si et al., 2021; Wong et al., 2021). At last, the filtered OD data were converted into HbO and HbR concentrations by applying the modified Beer–Lambert law.

#### 2.5.2 Regions of interest‐wised inter‐brain neural synchronization analysis

This study only focused on the HbO concentration due to its higher signal‐to‐noise compared to HbR (Mahmoudzadeh et al., 2013; Si et al., 2021). First, in order to analyze the changes of inter‐brain coupling as a whole, all the channels were divided into three ROIs including ROI_1 (PFC, CH1‐CH21, CH36), ROI_2 (r‐SMC, CH22‐CH28), and ROI_3 (l‐SMC, CH29‐CH35) according to the MNI coordinates.

Next, wavelet transform coherence (WTC) (Grinsted et al., 2004) was used to assess the relationship between the oxyhemoglobin time series from the corresponding ROIs of the two participants in each group (i.e., INS). WTC assesses the cross‐correlation between two time series as a function of time and frequency, which can reveal a locally phase‐locked behavior that may not be uncovered by traditional time series analysis such as Pearson's correlation(Torrence & Compo, 1998). The wavelet coherence MatLab package (Grinsted et al., 2004) was applied to calculate the INS in our frequency band of interest (i.e., 0.01–0.2 Hz). This frequency band can exclude the high‐ and low‐frequency noises, such as those associated with respiration (about 0.2–0.3 Hz) and cardiac pulsation (about 1 Hz), all of which would lead to artificial coherence. WTC analysis was conducted between the same ROIs of each dyad because shared representations of the same mental process were expected to be associated with INS at the same brain area (Dai et al., 2018). As the coherence value increases when there are interactions between individuals, compared with that during the baseline (Cui et al., 2012). The INS change was defined as the difference in INS between the interaction and rest/baseline session (i.e., INS_change_ = INS_interaction_ – INS_rest/baseline_). For each ROI, after converting the INS change value into Fisher *z*‐statistics, a one‐sample *t* test with false discovery rates (FDR) correction was performed on the *z* value across the dyads to identify significantly synchronized ROIs during acupuncture stimulation for two groups.

Finally, Pearson's correlation analysis was performed to further investigate the relationship between the significantly changed INS values of ROIs and needling sensation. Statistical analyses were conducted using MATLAB (version 2018a, MathWorks Inc.).

#### 2.5.3 Channel‐wised inter‐brain neural synchronization analysis

First, WTC was also applied in the channel‐wised INS analysis and a series of one‐sample *t* tests were conducted to find significantly synchronized channels during acupuncture stimuli for two groups. Then, independent‐sample *t* tests using group as the between‐subject factor were performed on group neural couplings. All resulting *p*‐values were corrected by the FDR method (*p* < .05).

For CHs that showed significant INS increments in each group, Granger causality analysis (GCA) was conducted to determine the direction of synchronization. GCA is a method that uses vector autoregressive models to measure the causal relationship between time series such as the fNIRS data and provides a neurobiological suggestion of coupling directionality, that is, which individual (the acupuncturist or the subject) is more actively driving the other. The HERMES MatLab package (http://hermes.ctb.upm.es) was employed to compute the pairwise‐conditional causalities of both directions: from the acupuncturists to the “patient” and from the “patient” to the acupuncturists. Then, one‐sample *t* tests were used to examine whether each direction differed from zero, and two‐sample *t* tests were applied to compare differences between two directions.

## 3 RESULTS

For schedule conflicts, three dyads (two in VAG, one in SAG) could not complete the experiment, and for poor quality of data, three dyads (two in VAG, one in SAG) could not be analyzed. Therefore, a total of 64 dyads (31 dyads in VAG and 33 dyads in SAG) were included in the final analyses.

There was no significant differences in age (VAG: 20.710 ± 2.341, SAG: 19.971 ± 1.360, *t*_(62)_ = 1.491, *p* = .141, Cohen's *d* = 0.379), BMI (VAG: 22.029 ± 3.377, SAG: 22.280 ± 3.040, *t*_(62)_ = −0.313, *p* = .756, Cohen's *d* = 0.078) and education years (VAG: 14.452 ± 2.241, SAG: 14.727 ± 1.675, *t*_(62)_ = −0.551, *p* = .584, Cohen's *d* = 0.139) between two groups. The score of needling sensations of VAG (25.065 ± 15.464) was significantly higher than that of SAG (5 ± 4.855, *t*_[62]_ = 7.073, *p* < .001, Cohen's *d* = 1.797).

### 3.1 The inter‐brain neural synchronization changes of regions of interests in both groups

**FIGURE 2:**
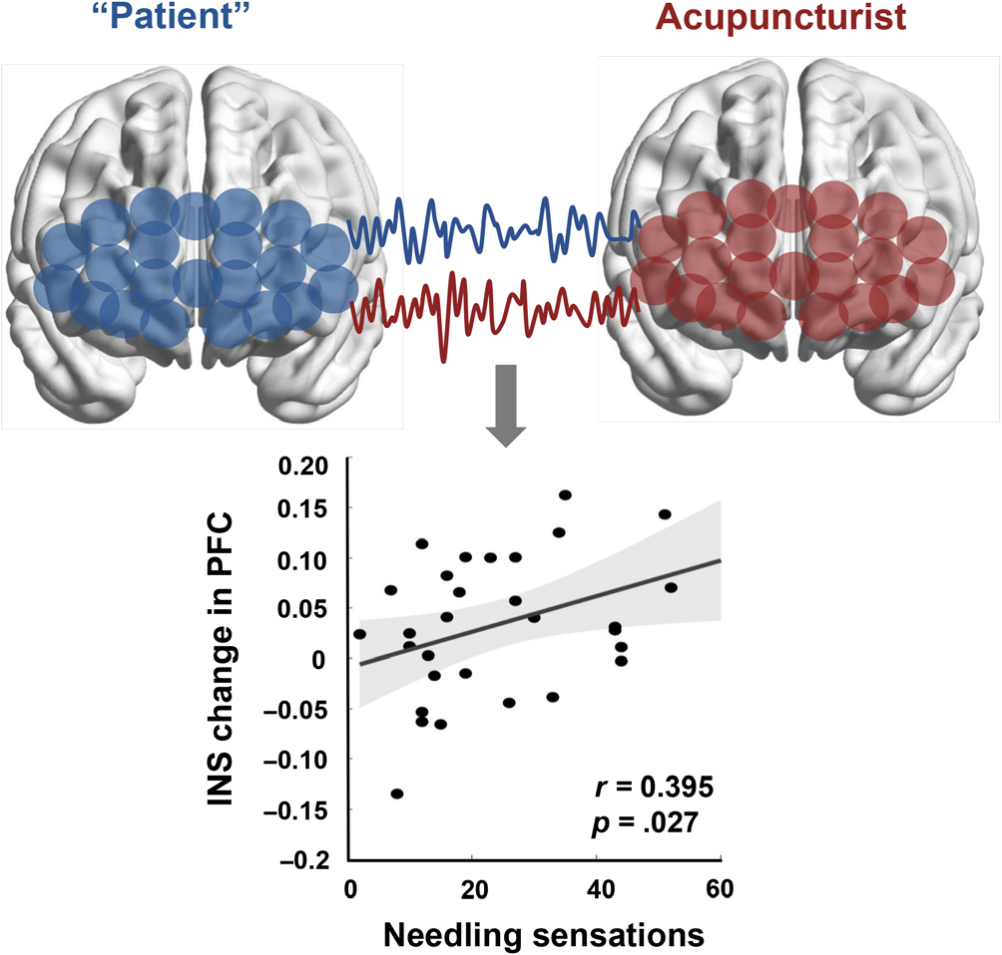
INS‐needling sensation correlation in “patient”–acupuncturist dyads. The blue and red curves represent the HbO time series from the ROI_1 of “patient” and acupuncturist, respectively. HbO, oxyhemoglobin; ROI, region of interest

For VAG, only ROI_1 (PFC, *t*_(30)_ = 2.814, *p*_(FDR)_ = .026, Cohen's *d* = 1.028) showed a significant INS increment during acupuncture manipulation. Pearson correlation analyses showed that INS increment of ROI_1 (PFC) was positively correlated with the needling sensations (C‐MASS) of VAG (*r* = 0.395, *p* = .028, Figure 2).

For SAG, no significant INS in any ROIs was found (*P* > .05, FDR corrected).

### 3.2 The inter‐brain neural synchronization change of channels at prefrontal cortex in both groups

**FIGURE 3:**
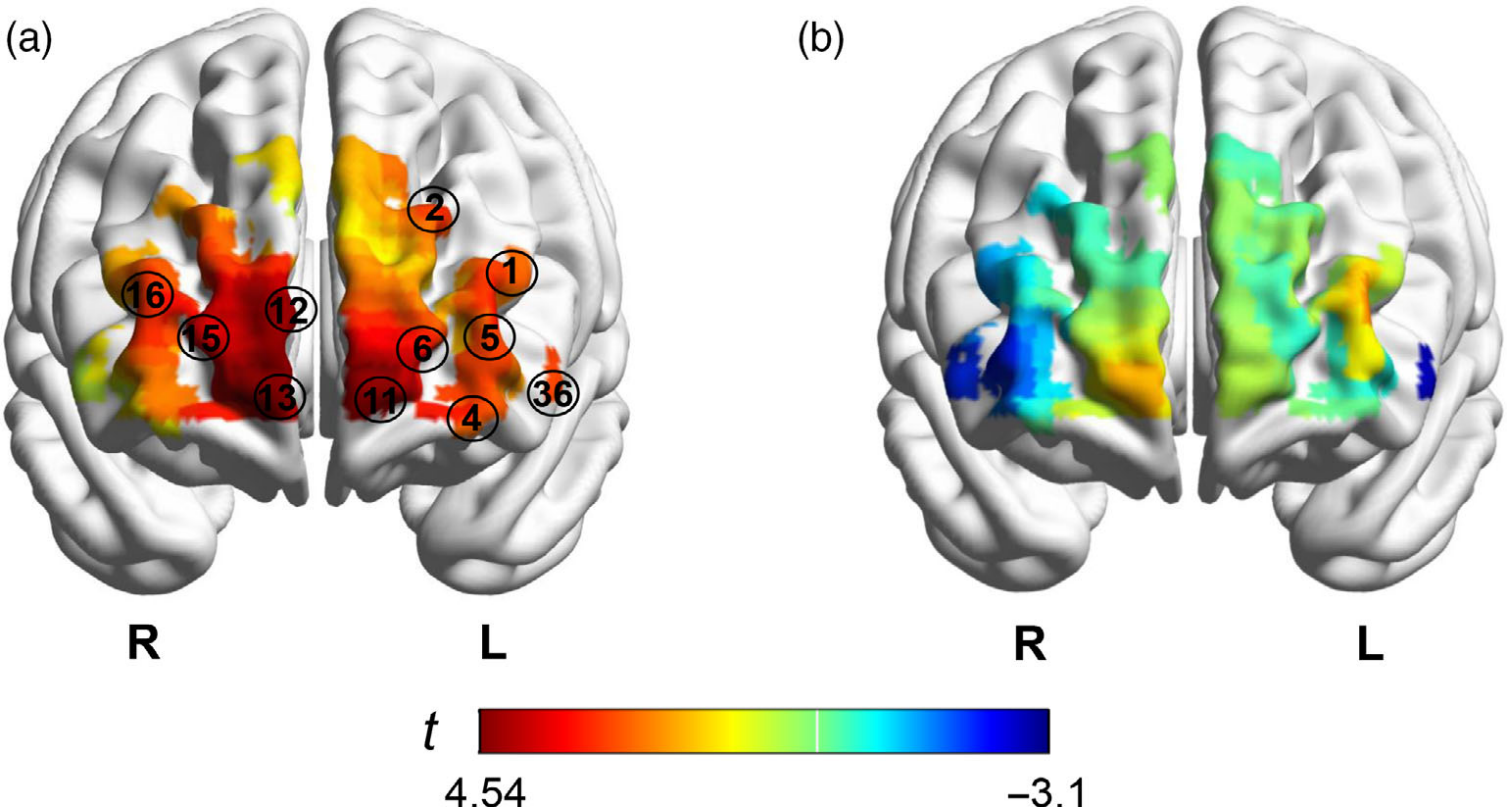
*T*‐value maps of acupuncture‐related INS of two groups. (a) Verum acupuncture group. (b) Sham acupuncture group. The number in black circle represents the channels with significantly increased task‐related INS. INS, inter‐brain neural synchronization

Compared with the baseline, the dyads in VAG demonstrated significant INS increment in 11 channels at PFC including the bilateral superior frontal cortex (SFC), middle frontal cortex (MFC) and left inferior frontal cortex (IFC) (Table 1 and Figure 3a).

**TABLE 1:** Acupuncture‐evoked INS in VAG

Channel	*t*_(30)_	*p*_(FDR)_	Cohen's *d*	Brain region
CH1	2.712	0.035	0.990	Left middle frontal gyrus
CH2	3.52	0.007	1.287	Left middle frontal gyrus
CH4	2.672	0.035	0.976	Left middle frontal gyrus
CH5	3.316	0.011	1.211	Left middle frontal gyrus
CH6	2.657	0.035	0.970	Left superior frontal gyrus
CH11	4.379	0.001	1.599	Left superior frontal gyrus
CH12	4.375	0.001	1.597	Right superior frontal gyrus
CH13	4.542	0.001	1.659	Right superior frontal gyrus
CH15	3.548	0.007	1.295	Right superior frontal gyrus
CH16	2.895	0.025	1.057	Right middle frontal gyrus
CH36	2.992	0.022	1.093	Left inferior frontal gyrus

Abbreviations: CH, channel; INS, inter‐brain neural synchronization; VAG, verum acupuncture group.

Furthermore, GCA was applied to explore the directionality of INS in 11 significant channels, finding that the mean G‐causalities of both directions were significantly higher than zero: from the subject to the acupuncturist (all *p* < .05, FDR corrected) and from the acupuncturist to the subject (all *p* < .05, FDR corrected). Whereas, there were no significant differences in coupling directionality between subject and acupuncturist (all *p* > .05, FDR corrected).

The dyads in SAG did not show significant changes of INS in all these channels (all *p* > .05, FDR corrected) (Figure 3b).

### 3.3 The between‐group differences in the inter‐brain neural synchronization of channels at prefrontal cortex

**FIGURE 4:**
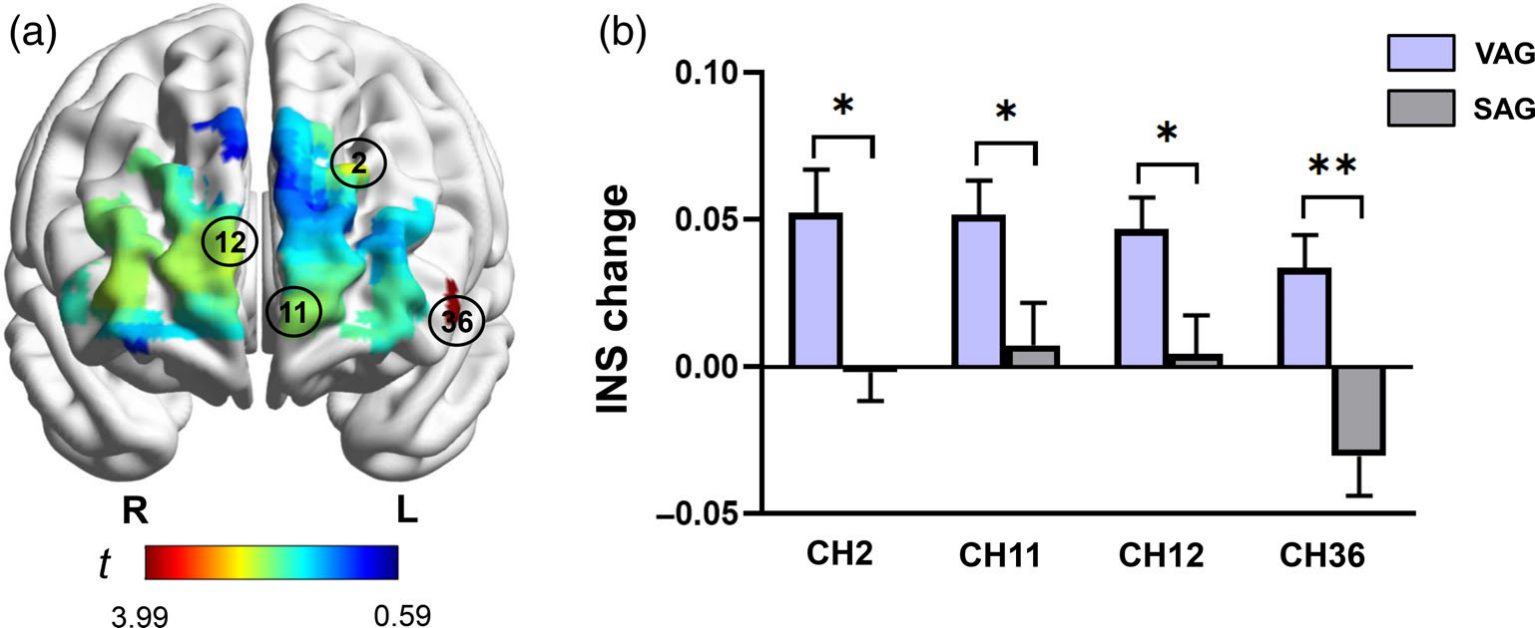
The between‐group differences in the INS at PFC. (a) The *t*‐map for independent‐samples *t* tests using group as the between‐subject factor on the INS increment of CH2, CH11, CH12, and CH36. (b) The amplitude of INS increment of the channels. Error bars indicate standard errors of the mean. *p < .05, **p < .01. CH, channel; INS, inter‐brain neural synchronization; PFC, prefrontal cortex; SAG, sham acupuncture group; VAG, verum acupuncture group

Independent‐sample *t* tests showed that compared with SAG, VAG showed significantly higher INS in four channels at PFC including the CH2 (VAG: 0.052 ± 0.082, SAG: −0.002 ± 0.056, *t*_(62)_ = 3.088, *p*_(FDR)_ = .020, Cohen's *d* = 0.774), CH11 (VAG: 0.051 ± 0.065, SAG: 0.007 ± 0.081, *t*_(62)_ = 2.373, *p*_(FDR)_ = .045, Cohen's *d* = 0.597), CH12 (VAG: 0.047 ± 0.059, SAG: 0.004 ± 0.073, *t*_(62)_ = 2.504, *p*_(FDR)_ = .039, Cohen's *d* = 0.646), and CH36 (VAG: 0.033 ± 0.062, SAG: −0.031 ± 0.077, *t*_(62)_ = 3.989, *p*_(FDR)_ = .003, Cohen's *d* = 0.912) (Figure 4).

## 4 DISCUSSION

This was the first fNIRS‐based hypersanning study to investigate the neurobiological mechanism of “patient”–acupuncturist interaction during acupuncture stimuli in a naturalistic clinical setting. The results demonstrated that INS within PFC (i.e., IFC, MFC, and SFC) of “patient”–acupuncturist dyad was significantly increased during verum but not sham acupuncture stimuli, and positively correlated with the needling sensations of “patients.”

### 4.1 Hyperscanning studies reveal the inter‐brain neural synchronization in patient–doctor interaction

As a new paradigm, hyperscanning realizes the conversion of traditional “one‐person” approach to “two or more person” approach and the transformation from brain–computer interaction to brain–brain coupling, which greatly enhanced the research on inter‐personal brain mechanism (Gvirts & Perlmutter, 2020; Schilbach et al., 2013). Recently, using hyperscanning techniques to reveal the influence of patient–doctor interaction on clinical outcomes attracts growing attention (Anzolin et al., 2020; Crum et al., 2022; Ellingsen et al., 2020; Ellingsen et al., 2022). For example, an fNIRS hyperscanning study reported that INS in the PFC of patient–doctor pair was significantly greater during clinical interactions compared to everyday‐life communication (Crum et al., 2022). INS is often observed between two or more brains during social interactions, which has been described as a mechanism of shared intentionality (Fishburn et al., 2018) that facilitates more attunement and greater allocation of attention to the interaction (Gvirts & Perlmutter, 2020). We suggested that INS in patient–doctor interaction may reflect the mindset synchrony between patient and doctor and greater INS may lead to more tuning in with one another and the interaction's goals, facilitating greater mutual attention.

### 4.2 Prefrontal cortex is the key region involved in the patient–acupuncturist interaction

This study found that the increased INS was mostly present over the PFC including SFC, MFC, and IFC, and positively correlated with the needling sensation. PFC is critical for top‐down regulation of attention, emotion, decision‐making, and higher cognitive functions (Arnsten & Rubia, 2012; Minati et al., 2012). Importantly, PFC is closely linked with the theory of mind (ToM) which refers to the ability to attribute mental states to self and others, including knowledge, beliefs and intentions (Sebastian et al., 2012). The IFC is viewed as an important hub of the mirror neuron system (Iacoboni & Mazziotta, 2007), proposed to facilitate social interaction by predicting other individuals' actions and intentions(Gallese, 2013). So, the increased INS in PFC may suggest that during the acupuncture treatment, both patient and acupuncturist should concentrate their mind on the acupuncture manipulation and perceive needling sensations. The acupuncturist should pay attention to the patient's responses, and accurately capture the sensory changes from his/her fingers (which hold the needle) to determine the course of further manipulation. Meanwhile, the patient should carefully experience the changes of local sensation induced by acupuncture stimulation. Based on fMRI hyperscanning, Ellingsen and colleagues also found that INS in PFC was significantly enhanced for patient–acupuncturist dyad during acupuncture treatment (Ellingsen et al., 2020). Thus, PFC may serve a key role in mediating patient–acupuncturist interaction behavior and brain–brain coupling.

In this study, the INS increments were found within PFC of “patient”–acupuncturist dyad, and significantly related to the needling sensation. The results indicated that needling sensations may have contributed to the increase of INS between “patient” and acupuncturist. Compared to the “patient” in SAG, the “patient” in VAG experience stronger, much complex and lasting needling sensations. At the same time, the acupuncturist also reported stronger needling sensations from her fingers holding the needle when performing verum acupuncture rather than sham acupuncture manipulation. In fact, the acupuncturist did not feel any needling sensations when performing the sham acupuncture manipulation. This deeper and stronger verum acupuncture stimulation elicits more attention from both “patient” and acupuncturist, and activates deeper, sub‐dermal receptors, etc., which in turn leads to more significant emotional, cognitive, and interoceptive processing (Napadow et al., 2009). This may be the reason why non‐invasive sham acupuncture without obvious needling sensations did not induce significantly increased INS in the “patient”–acupuncturist pair. As needling sensation is the key to obtaining acupuncture effect, we speculated that there may be a certain relationship between INS of “patient”–acupuncturist dyad and acupuncture effect. Future studies are encouraged to detect whether INS in PFC of patient–acupuncturist pair is a potential neurobiological marker to predict the treatment outcome.

In the current study, channel‐wised INS analysis indicated that 11 channels at the PFC showed significantly increased INS in the VAG, and none of all the channels in SAG showed significant changes of INS. These findings suggested that PFC is an important region involved in the “patient”–acupuncturist interaction during acupuncture stimulation. INS in PFC might support interpersonal information transfer by aligning neural processes in the “patient”–acupuncturist dyad.

In order to further examine this interpretation, GCA was used to determine whether brain activity recorded from the “patient” could be predicted by that recorded from the acupuncturist. Interestingly, GCA demonstrated that there were no significant differences in coupling directionality between the “patient” and the acupuncturist. Despite the common belief that the acupuncturist plays a more predominant and active role in acupuncture treatment (Liu, 2007), these results may imply that the involvement of the patient and acupuncturist in acupuncture treatment is actually equal. In other words, the acupuncture treatment requires the cooperation of both patient and acupuncturist rather than the dominance of either. Certainly, the coupling directionality between patient and acupuncturist is of great interest and needs additional research.

### 4.3 Limitations and future direction

This study should be reviewed with these two limitations. First, healthy subjects, not patients, were included in this study, which made it hard to analyze the impact of patient–acupuncturist interaction on therapeutic effects. Therefore, this study chose the needling sensation which is the key to obtain acupuncture effect as an “indicator” of acupuncture effect. Secondly, only one acupuncturist was recruited in this study to ensure standardized acupuncture manipulation, while the experience of acupuncturists might affect the patient–acupuncturist interaction. Future study could consider enrolling patients with recurrent disorders as well as acupuncturists with different years of clinical experience to further explore the relationship between INS of patient–acupuncturist dyad and acupuncture effect and to detect the influence of acupuncturist's experience on the INS of patient–acupuncturist dyad.

In summary, this study first applied fNIRS hyperscanning to investigate the neurobiological mechanism of “patient”–acupuncturist interaction during acupuncture stimuli in a naturalistic clinical setting. The results identified the increase of INS between “patient” and acupuncturist, and found that PFC was important to the interaction of “patient”–acupuncturist. This study deepen our understanding on the patient–acupuncturist interaction, and provided a novel experimental approach for future studies on acupuncture mechanism.

## AUTHOR CONTRIBUTION

Li Chen: Conceptualization, Methodology, Formal analysis, Investigation, Data curation, Writing‐original draft. Yuzhu Qu: Investigation, Data curation, Formal analysis, Writing – review & editing. Jingya Cao: Investigation, Data curation, Methodology, Writing – review & editing. Tianyu Liu: Investigation, Resources. Yulai Gong: Investigation, Resources. Zilei Tian: Investigation, Software. Jing Xiong: Investigation. Zhenfang Lin: Investigation. Xin Yang: Investigation, Resources. Tao Yin: Conceptualization, Methodology, Software, Supervision, Writing – review & editing. Fang Zeng: Conceptualization, Methodology, Funding acquisition, Supervision, Writing – review & editing.

## FUNDING INFORMATION

This study is financially supported by the National Science Fund for Distinguished Young Scholars (82225050), National Natural Science Foundation of China (81973960 and 82205285), Sichuan Scientific and Technological Innovation Team (2019JDTD0011) and China Postdoctoral Science Foundation (2021M700552).

## CONFLICT OF INTEREST

The authors have no conflicts of interest to declare.

## CLINICAL TRIAL REGISTRATION

This study was registered in the Chinese Clinical Trial Registry on October 9, 2021 (Registered number: ChiCTR2100051893).

## Supporting information


**Table S1:** Average coordinates, anatomical regions, and atlas probabilities of channels

## Data Availability

The raw fNIRS data of our study may be made available upon request after a confirmation from the ethical committee of our institution.

## References

[hbm26120-bib-0001] Anzolin, A. , Isenburg, K. , Grahl, A. , Toppi, J. , Yucel, M. , Ellingsen, D. M. , Gerber, J. , Ciaramidaro, A. , Astolfi, L. , Kaptchuk, T. J. , & Napadow, V. (2020). Patient‐clinician brain response during clinical encounter and pain treatment. Annual International Conference of the IEEE Engineering in Medicine and Biology Society, 2020, 1512–1515. 10.1109/embc44109.2020.9175608 33018278 PMC8096120

[hbm26120-bib-0002] Arnsten, A. F. , & Rubia, K. (2012). Neurobiological circuits regulating attention, cognitive control, motivation, and emotion: Disruptions in neurodevelopmental psychiatric disorders. Journal of the American Academy of Child and Adolescent Psychiatry, 51, 356–367. 10.1016/j.jaac.2012.01.008 22449642

[hbm26120-bib-0003] Bishop, F. , Al‐Abbadey, M. , Roberts, L. , Macpherson, H. , Stuart, B. , Carnes, D. , Fawkes, C. , Yardley, L. , & Bradbury, K. (2021). Direct and mediated effects of treatment context on low back pain outcome: A prospective cohort study. BMJ Open, 11, e044831. 10.1136/bmjopen-2020-044831 PMC8130743 34006548

[hbm26120-bib-0004] Crum, J. , Zhang, X. , Noah, A. , Hamilton, A. , Tachtsidis, I. , Burgess, P. , & Hirsch, J. (2022). An approach to neuroimaging interpersonal interactions in mental health interventions. Biological Psychiatry: Cognitive Neuroscience and Neuroimaging, 7, 669–679. 10.1016/j.bpsc.2022.01.008 35144035 PMC9271588

[hbm26120-bib-0005] Cui, X. , Bryant, D. M. , & Reiss, A. L. (2012). NIRS‐based hyperscanning reveals increased interpersonal coherence in superior frontal cortex during cooperation. NeuroImage, 59, 2430–2437. 10.1016/j.neuroimage.2011.09.003 21933717 PMC3254802

[hbm26120-bib-0006] Dai, B. , Chen, C. , Long, Y. , Zheng, L. , Zhao, H. , Bai, X. , Liu, W. , Zhang, Y. , Liu, L. , Guo, T. , Ding, G. , & Lu, C. (2018). Neural mechanisms for selectively tuning in to the target speaker in a naturalistic noisy situation. Nature Communications, 9, 2405. 10.1038/s41467-018-04819-z PMC6008393 29921937

[hbm26120-bib-0007] Ellingsen, D. M. , Duggento, A. , Isenburg, K. , Jung, C. , Lee, J. , Gerber, J. , Mawla, I. , Sclocco, R. , Edwards, R. R. , Kelley, J. M. , Kirsch, I. , Kaptchuk, T. J. , Toschi, N. , & Napadow, V. (2022). Patient‐clinician brain concordance underlies causal dynamics in nonverbal communication and negative affective expressivity. Translational Psychiatry, 12, 44. 10.1038/s41398-022-01810-7 35091536 PMC8799700

[hbm26120-bib-0008] Ellingsen, D. M. , Isenburg, K. , Jung, C. , Lee, J. , Gerber, J. , Mawla, I. , Sclocco, R. , Jensen, K. B. , Edwards, R. R. , Kelley, J. M. , Kirsch, I. , Kaptchuk, T. J. , & Napadow, V. (2020). Dynamic brain‐to‐brain concordance and behavioral mirroring as a mechanism of the patient‐clinician interaction. Science Advances, 6, eabc1304. 10.1126/sciadv.abc1304 33087365 PMC7577722

[hbm26120-bib-0009] Fei, Y. T. , Cao, H. J. , Xia, R. Y. , Chai, Q. Y. , Liang, C. H. , Feng, Y. T. , Du, Y. R. , Yu, M. K. , Guyatt, G. , Thabane, L. , Lao, L. X. , Liu, J. P. , & Zhang, Y. Q. (2022). Methodological challenges in design and conduct of randomised controlled trials in acupuncture. British Medical Journal, 376, e064345. 10.1136/bmj-2021-064345 35217507 PMC8868049

[hbm26120-bib-0010] Fernandez Rojas, R. , Liao, M. , Romero, J. , Huang, X. , & Ou, K. L. (2019). Cortical network response to acupuncture and the effect of the Hegu point: An fNIRS study. Sensors (Basel), 19, 394. 10.3390/s19020394 30669377 PMC6359459

[hbm26120-bib-0011] Fishburn, F. A. , Murty, V. P. , Hlutkowsky, C. O. , Macgillivray, C. E. , Bemis, L. M. , Murphy, M. E. , Huppert, T. J. , & Perlman, S. B. (2018). Putting our heads together: Interpersonal neural synchronization as a biological mechanism for shared intentionality. Social Cognitive and Affective Neuroscience, 13, 841–849. 10.1093/scan/nsy060 30060130 PMC6123517

[hbm26120-bib-0012] Gallese, V. (2013). Mirror neurons, embodied simulation and a second‐person approach to mindreading. Cortex, 49, 2954–2956. 10.1016/j.cortex.2013.09.008 24209736

[hbm26120-bib-0013] Ghafoor, U. , Lee, J. H. , Hong, K. S. , Park, S. S. , Kim, J. , & Yoo, H. R. (2019). Effects of acupuncture therapy on MCI patients using functional near‐infrared spectroscopy. Frontiers in Aging Neuroscience, 11, 237. 10.3389/fnagi.2019.00237 31543811 PMC6730485

[hbm26120-bib-0014] Grinsted, A. , Moore, J. C. , & Jevrejeva, S. (2004). Application of the cross wavelet transform and wavelet coherence to geophysical time series. Nonlinear Processes in Geophysics, 11, 561–566. 10.5194/npg-11-561-2004

[hbm26120-bib-0015] Gvirts, H. Z. , & Perlmutter, R. (2020). What guides us to Neurally and behaviorally align with anyone specific? A neurobiological model based on fNIRS Hyperscanning studies. The Neuroscientist, 26, 108–116. 10.1177/1073858419861912 31296135

[hbm26120-bib-0016] Hamilton, A. F. C. (2021). Hyperscanning: Beyond the hype. Neuron, 109, 404–407. 10.1016/j.neuron.2020.11.008 33259804

[hbm26120-bib-0017] Hoshi, Y. (2007). Functional near‐infrared spectroscopy: Current status and future prospects. Journal of Biomedical Optics, 12, 062106. 10.1117/1.2804911 18163809

[hbm26120-bib-0018] Hoshi, Y. (2016). Hemodynamic signals in fNIRS. Progress in Brain Research, 225, 153–179. 10.1016/bs.pbr.2016.03.004 27130415

[hbm26120-bib-0019] Iacoboni, M. , & Mazziotta, J. C. (2007). Mirror neuron system: Basic findings and clinical applications. Annals of Neurology, 62, 213–218. 10.1002/ana.21198 17721988

[hbm26120-bib-0020] Liu, T. (2007). Role of acupuncturists in acupuncture treatment. Evidence‐based Complementary and Alternative Medicine, 4, 3–6. 10.1093/ecam/nel061 PMC1810358 17342235

[hbm26120-bib-0021] Mahmoudzadeh, M. , Dehaene‐Lambertz, G. , Fournier, M. , Kongolo, G. , Goudjil, S. , Dubois, J. , Grebe, R. , & Wallois, F. (2013). Syllabic discrimination in premature human infants prior to complete formation of cortical layers. Proceedings of the National Academy of Sciences of the United States of America, 110, 4846–4851. 10.1073/pnas.1212220110 23440196 PMC3607062

[hbm26120-bib-0022] Mawla, I. , Ichesco, E. , Zöllner, H. J. , Edden, R. , A. E. , Chenevert, T. , Buchtel, H. , Bretz, M. D. , Sloan, H. , Kaplan, C. M. , Harte, S. E. , Mashour, G. A. , Clauw, D. J. , Napadow, V. , & Harris, R. E. (2021). Greater somatosensory Afference with acupuncture increases primary somatosensory connectivity and alleviates fibromyalgia pain via insular γ‐aminobutyric acid: A randomized neuroimaging trial. Arthritis & Rhematology, 73, 1318–1328. 10.1002/art.41620 PMC8197768 33314799

[hbm26120-bib-0023] Minati, L. , Campanhã, C. , Critchley, H. D. , & Boggio, P. S. (2012). Effects of transcranial direct‐current stimulation (tDCS) of the dorsolateral prefrontal cortex (DLPFC) during a mixed‐gambling risky decision‐making task. Cognitive Neuroscience, 3, 80–88. 10.1080/17588928.2011.628382 24168688

[hbm26120-bib-0024] Napadow, V. , Dhond, R. P. , Kim, J. , Lacount, L. , Vangel, M. , Harris, R. E. , Kettner, N. , & Park, K. (2009). Brain encoding of acupuncture sensation‐coupling on‐line rating with fMRI. NeuroImage, 47, 1055–1065. 10.1016/j.neuroimage.2009.05.079 19500677 PMC2733781

[hbm26120-bib-0025] Schilbach, L. , Timmermans, B. , Reddy, V. , Costall, A. , Bente, G. , Schlicht, T. , & Vogeley, K. (2013). Toward a second‐person neuroscience. Behavioral and Brain Sciences, 36, 393–414. 10.1017/s0140525x12000660 23883742

[hbm26120-bib-0026] Sebastian, C. L. , Fontaine, N. M. , Bird, G. , Blakemore, S. J. , Brito, S. A. , Mccrory, E. J. , & Viding, E. (2012). Neural processing associated with cognitive and affective theory of mind in adolescents and adults. Social Cognitive and Affective Neuroscience, 7, 53–63. 10.1093/scan/nsr023 21467048 PMC3252629

[hbm26120-bib-0027] Si, X. , Xiang, S. , Zhang, L. , Li, S. , Zhang, K. , & Ming, D. (2021). Acupuncture with deqi modulates the hemodynamic response and functional connectivity of the prefrontal‐motor cortical network. Frontiers in Neuroscience, 15, 693623. 10.3389/fnins.2021.693623 34483822 PMC8415569

[hbm26120-bib-0028] Suarez‐Almazor, M. E. , Looney, C. , Liu, Y. , Cox, V. , Pietz, K. , Marcus, D. M. , & Street, R. L., Jr. (2010). A randomized controlled trial of acupuncture for osteoarthritis of the knee: Effects of patient‐provider communication. Arthritis Care and Research (Hoboken), 62, 1229–1236. 10.1002/acr.20225 PMC3651275 20506122

[hbm26120-bib-0029] Takamoto, K. , Hori, E. , Urakawa, S. , Sakai, S. , Ishikawa, A. , Kohno, S. , Ono, T. , & Nishijo, H. (2010). Cerebral hemodynamic responses induced by specific acupuncture sensations during needling at trigger points: A near‐infrared spectroscopic study. Brain Topography, 23, 279–291. 10.1007/s10548-010-0148-8 20502956

[hbm26120-bib-0030] Torrence, C. , & Compo, G. P. (1998). A practical guide to wavelet analysis. Bulletin of the American Meteorological Society, 79, 61–78. 10.1175/1520-0477(1998)079<0061:apgtwa>2.0.co;2

[hbm26120-bib-0031] Wang, Q. , Zhu, G. P. , Yi, L. , Cui, X. X. , Wang, H. , Wei, R. Y. , & Hu, B. L. (2020). A review of functional near‐infrared spectroscopy studies of motor and cognitive function in preterm infants. Neuroscience Bulletin, 36, 321–329. 10.1007/s12264-019-00441-1 31713716 PMC7056771

[hbm26120-bib-0032] Wong, Y. K. , Wu, J. M. , Zhou, G. , Zhu, F. , Zhang, Q. , Yang, X. J. , Qin, Z. , Zhao, N. , Chen, H. , & Zhang, Z. J. (2021). Antidepressant monotherapy and combination therapy with acupuncture in depressed patients: A resting‐state functional near‐infrared spectroscopy (fNIRS) study. Neurotherapeutics, 18, 2651–2663. 10.1007/s13311-021-01098-3 34431029 PMC8804104

[hbm26120-bib-0033] Yu, D. T. , Jones, A. Y. , & Pang, M. Y. (2012). Development and validation of the Chinese version of the Massachusetts General Hospital acupuncture sensation scale: An exploratory and methodological study. Acupuncture in Medicine, 30, 214–221. 10.1136/acupmed-2012-010145 22617434

[hbm26120-bib-0034] Zhang, Y. Q. , Jing, X. , & Guyatt, G. (2022). Improving acupuncture research: Progress, guidance, and future directions. British Medical Journal, 376, o487. 10.1136/bmj.o487 35217523

