# Supplementary material for: The increased inter‐brain neural synchronization in prefrontal cortex between simulated patient and acupuncturist during acupuncture stimulation: Evidence from functional near‐infrared spectroscopy hyperscanning
Source: Hum Brain Mapp. 2022 Oct 18;44(3):980–8. doi: 10.1002/hbm.26120 (PMC9875919; doi:10.1002/hbm.26120)
Supplement: Supplementary file 1 — Table S1: Average coordinates, anatomical regions, and atlas probabilities of channels [file HBM-44-980-s001.docx]

**Supplementary Table Average Coordinates, Anatomical Regions, and Atlas Probabilities of Channels**

| **CH** | **MNI coordinates** | | | **Anatomical Region** | **Probability** |
| --- | --- | --- | --- | --- | --- |
|  | **X** | **Y** | **Z** |  |  |
| **1** | -47 | 43 | 24 | Left Middle Frontal Gyrus-BA45 | 0.75 |
| **2** | -30 | 46 | 42 | Left Middle Frontal Gyrus-BA9 | 0.54 |
| **3** | -49 | 49 | -1 | Left Inferior Frontal Gyrus-BA46 | 0.32 |
| **4** | -34 | 64 | -9 | Left Middle Frontal Gyrus-BA11 | 0.79 |
| **5** | -42 | 55 | 15 | Left Middle Frontal Gyrus-BA46 | 0.93 |
| **6** | -26 | 68 | 4 | Left Superior Frontal Gyrus-BA10 | 0.76 |
| **7** | -14 | 68 | 24 | Left Superior Frontal Gyrus-BA10 | 0.69 |
| **8** | -10 | 46 | 52 | Left Superior Frontal Gyrus-BA9 | 0.53 |
| **9** | 1 | 55 | 41 | Left Superior Frontal Gyrus-BA9 | 0.63 |
| **10** | 13 | 46 | 53 | Right Superior Frontal Gyrus-BA9 | 0.53 |
| **11** | -11 | 73 | -5 | Left Superior Frontal Gyrus-BA11 | 0.38 |
| **12** | 3 | 69 | 13 | Right Superior Frontal Gyrus-BA10 | 0.50 |
| **13** | 14 | 73 | -5 | Right Superior Frontal Gyrus-BA11 | 0.41 |
| **14** | 17 | 68 | 25 | Right Superior Frontal Gyrus-BA10 | 0.60 |
| **15** | 29 | 69 | 5 | Right Superior Frontal Gyrus-BA10 | 0.74 |
| **16** | 45 | 55 | 16 | Right Middle Frontal Gyrus-BA46 | 1 |
| **17** | 34 | 45 | 43 | Right Middle Frontal Gyrus-BA9 | 0.81 |
| **18** | 50 | 43 | 26 | Right Middle Frontal Gyrus-BA45 | 0.79 |
| **19** | 37 | 65 | -10 | Right Middle Frontal Gyrus-BA11 | 0.73 |
| **20** | 52 | 48 | 0 | Right Inferior Frontal Gyrus-BA46 | 0.30 |
| **21** | 57 | 38 | 1 | Right Inferior Frontal Gyrus-BA45 | 0.62 |
| **22** | 65 | -18 | 44 | Right Primary Somatosensory Cortex-BA1 | 0.54 |
| **23** | 55 | -32 | 58 | Right Primary Somatosensory Cortex-BA1 | 0.44 |
| **24** | 44 | -16 | 68 | Right Primary Motor Cortex-BA4 | 0.59 |
| **25** | 70 | -35 | 29 | Right Primary Somatosensory Cortex-BA1 | 0.26 |
| **26** | 62 | -49 | 46 | Right Supramarginal Gyrus-BA40 | 0.92 |
| **27** | 40 | -48 | 68 | Right Somatosensory Association Cortex-BA7 | 0.39 |
| **28** | 29 | -31 | 75 | Right Primary Motor Cortex-BA4 | 0.69 |
| **29** | -62 | -48 | 45 | Left Supramarginal Gyrus-BA40 | 0.96 |
| **30** | -69 | -34 | 27 | Left Primary Somatosensory Cortex-BA1 | 0.34 |
| **31** | -29 | -31 | 74 | Left Primary Motor Cortex-BA4 | 0.72 |
| **32** | -40 | -47 | 68 | Left Somatosensory Association Cortex-BA7 | 0.28 |
| **33** | -43 | -16 | 67 | Left Primary Motor Cortex-BA4 | 0.62 |
| **34** | -54 | -31 | 57 | Left Primary Somatosensory Cortex-BA1 | 0.41 |
| **35** | -64 | -17 | 42 | Left Primary Somatosensory Cortex-BA1 | 0.40 |
| **36** | -55 | 38 | -1 | Left Inferior Frontal Gyrus-BA45 | 0.76 |
